# Factors Associated with RANTES Concentration in Cardiovascular Disease Patients

**DOI:** 10.1155/2019/3026453

**Published:** 2019-07-18

**Authors:** Olga M. Koper-Lenkiewicz, Joanna Kamińska, Anna Lisowska, Anna Milewska, Tomasz Hirnle, Violetta Dymicka-Piekarska

**Affiliations:** ^1^Department of Clinical Laboratory Diagnostics, Medical University of Bialystok, Poland; ^2^Department of Cardiology, Medical University of Bialystok, Bialystok, Poland; ^3^Department of Statistics and Medical Informatics, Medical University of Bialystok, Poland; ^4^Department of Cardiosurgery, Medical University of Bialystok, Poland

## Abstract

**Objective:**

The aim of the study was to establish, by means of linear regressions analysis, whether RANTES and CCL2 have a relationship with age, sex, heart rate, ejection fraction, white blood cells count, monocyte count, platelet count, mean platelet volume, hsCRP concentration, creatinine and eGFR value, applied treatments, and coronary risk factors in polish cardiovascular disease patients.

**Methods:**

Plasma chemokines concentrations were measured by ELISA method (R&D Systems Europe Ltd., Abingdon, England) in 115 cardiovascular disease patients (83 myocardial infarction/AMI and 32 stable angina/SA) and in the control group (N=25).

**Results:**

Univariate linear regression analysis found that (1) for men mean RANTES plasma level is 1.56 times higher as compared to women; (2) if patient's age increases by 1 year, the mean RANTES concentration value increases by 1.4%; (3) if CCL2 concentration increases by 10 pg/mL, the mean RANTES concentration value increases by 3.3%; (4) if hsCRP concentration increases by 1 mg/L, the mean RANTES concentration value increases by 1.0%. By means of multiple linear regression analysis we found that (1) for men the mean plasma RANTES concentration value increases 1.89 times as compared to women; (2) if CCL2 concentration increases by 10 pg/mL, the mean RANTES concentration value increases by 3.4%; (3) if MPV increases by 1 fL, the mean RANTES concentration value increases by 12%, if other model parameters are fixed. For CCL2 we did not obtain statistically significant linear regression models.

**Conclusion:**

Due to high variability of obtained CCL2 concentrations, it seems that RANTES better reflects the presence of the atherosclerotic lesion than CCL2. RANTES as a marker of atherosclerotic process may be an important therapeutic target, and the assessment of RANTES concentration should be interpreted depending on patient's sex, age, platelet hyperactivity state, hsCRP, and CCL2 concentration.

## 1. Introduction

In the last decades experimental studies reported that chemokines can have a role in the pathophysiology of cardiovascular disease (CVD) [1–4]. It currently seems that the inhibition of chemokine-mediated recruitment and activation of monocytes during atherosclerotic plaque formation may present a novel therapeutic target to counteract the development and progression of coronary artery disease (CAD) [5].

CC chemokine engaged in the pathophysiology of cardiovascular disease is RANTES (Regulated upon Activation, Normal T cell Expressed and presumably Secreted), which is expressed by different cell types, e.g., T cells, fibroblasts, and some kinds of tissue monocytes [6–8]. It is responsible for the two main stages of atherogenesis: leukocytes chemotaxis onto the endothelial wall and induction of transendothelial migration of leukocytes. RANTES is also stored in *α*-granules of the platelets [7, 8] and deposited on the surface of damaged endothelial cells after platelet activation [7]. This chemokine is considered a key player of the process, in which activated platelets support and maintain atherogenic recruitment of monocytes, which may accelerate the atherosclerotic plaque formation.

CCL2, also known as MCP-1 (monocyte chemoattractant protein 1) belongs to the CC chemokines especially involved in the pathogenesis of cardiovascular disease, which was well established in animal models [9–12]. It is engaged in monocyte/macrophages, T cells, and NK cells recruitment and activation on the site of inflammation [13]. In apolipoprotein E-deficient mice, CCL2 accelerated atherosclerotic plaque formation [9]. Moreover, studies revealed attenuated macrophages accumulation in the aorta of CCL-2-deficient mice [10].

The knowledge about circulating concentrations of chemokines RANTES and CCL2 in cardiovascular disease patients is still insufficiently documented, so better understanding of variables that may influence their levels is vital to the field, especially that the inhibition of chemokine-mediated recruitment and activation of monocytes may present a novel therapeutic target to counteract the development and progression of atherosclerotic plaque formation.

Therefore the aim of current study was to evaluate the concentrations of chemokines RANTES and CCL2 in acute myocardial infarction (AMI) patients and stable angina (SA) subjects on their admission to the hospital, as compared to healthy control subjects. In the next step, we tried to establish whether RANTES and CCL2 have a relationship with age, sex, heart rate, ejection fraction, white blood cells count (WBC), monocyte count, platelet count (PLT), mean platelet volume (MPV), high sensitivity C-reactive protein (hsCRP) concentration, creatinine and eGFR value, applied treatments (aspirin, heparin, and clopidogrel), and coronary risk factors (lipid profile parameters, glucose concentration, blood pressure, obesity, and smoking).

## 2. Material and Methods

The study was conducted in agreement with the Helsinki-II Declaration and was approved by the Bioethics Human Research Committee of the Medical University of Bialystok (permission number: R-I-002/355/2017). All subjects included in the study gave their written informed consent.

### 2.1. Study Group

The study group was composed of 115 patients hospitalized between 2015 and 2016 in the Department of Cardiology and the Department of Cardiosurgery, divided into acute myocardial infarction subjects (AMI) (N=83) and stable angina (SA) (N=32). The AMI group was divided according to the clinical diagnosis into two subgroups: 38 patients with ST-elevation myocardial infarction (STEMI) and 45 patients with non-ST-elevation myocardial infarction (NSTEMI).

The study group inclusion criteria were patient's age between 18 and 80 years old, a diagnosed myocardial infarction, and a completed coronary angiography. The number of subjects included in each group resulted from the exclusion criteria: pulmonary edema, severe renal failure (5^th^ stage according to NKF), and cardiogenic shock.

The AMI patients underwent invasive procedures: coronary angiography (a significant lesion was defined as lumen stenosis of more than 50%) and angioplasty in the acute phase of myocardial infarction. The criteria to diagnose myocardial infarction included the reference standards: the detection of rise or fall of necrosis cardiac biomarker (preferably high sensitive troponin I with at last one value above the 99^th^ percentile URL) and at least one of the following: clinical symptoms of ischemia, new or presumed new significant ST-segment-T wave changes or new LBBB, the development of pathological Q waves, imaging evidence of a new loss of variable myocardium, or a new regional wall motion abnormality.

The SA group included patients who, due to reported chest pain, were referred by a GP to the Cardiology Clinic for further diagnosis and implementation or optimization of pharmacotherapy. Based on the noninvasive stress tests (treadmill test or stress echocardiography) resulting SA patients were assigned to coronary angiography (a significant lesion was defined as lumen stenosis of more than 50%) and after the consultation with the heart team were qualified for invasive treatment of coronary artery disease, PCI or CABG.

In the AMI group 30 patients (36%) had the 1-vessel disease, and the remaining 53 patients (53%) had the ≥ 2-vessel disease. In the STEMI subgroup 16 patients (42%) had the 1-vessel disease, and the remaining 22 patients (58%) had the ≥ 2-vessel disease. In the NSTEMI subgroup 14 patients (31%) had the 1-vessel disease, and the remaining 31 patients (69%) had the ≥ 2-vessel disease. In the SA group 7 patients (22%) had the 1-vessel disease, and the remaining 25 patients (78%) had the ≥ 2-vessel disease.  Table 1 presents clinical characteristics of the cardiovascular disease patients (Table 1).

### 2.2. Control Group

The control group included 25 healthy subjects (8 females/17 males; median age 66 years, range 52-74 years) undergoing their periodical check-ups at the Medical University Hospital's Clinic. Their medical history did not reveal any cardiovascular problems, diabetes, or hypertension. Routinely performed ECG was normal, and the blood pressures values were within normal ranges. They also had no abnormalities in concentrations of lipids profile (total cholesterol, LDL-cholesterol, HDL-cholesterol, triglycerides), creatinine and GFR (glomerular filtration rate), and glucose. The exclusion criteria for the control group were infections, inflammations, any systemic diseases, and renal failure. Controls also did not take anti-inflammatory or any antiplatelet drugs during last 7-10 days.  Table 2 presents laboratory findings of cardiovascular disease patients in comparison to the control group (Table 2).

### 2.3. RANTES and CCL2 Evaluation

Venous blood samples were drawn on patient's admission to the hospital. In order to perform RANTES and CCL2 examinations, blood (3.0 mL) was collected into the test tubes S-Monovette (SARSTEDT) containing 3.2% sodium citrate (1:9, v:v) as an anticoagulant and centrifuged within 30 minutes of collection. Plasma samples for RANTES evaluation were obtained via double centrifugation: first for 10 min at 1000 x g and second for 10 min at 10 000 x g (in 4°C temperature) to remove platelets and obtain poor platelet plasma (PPP). The PPP samples were separated and stored at -75°C until assay. Plasma samples for CCL2 evaluation were collected by centrifugation of blood samples for 15 min at 1000 x g. The plasma samples were separated and stored at -75°C until assay.

Prior to the assay, all plasma samples were gradually defrosted and mixed using a Vortex. Plasma concentrations of RANTES and CCL2 were quantified by means of ELISA test using Quantikine Human Immunoassays (R&D Systems Europe Ltd., Abingdon, England). This assay employs a quantitative sandwich enzyme-linked immunosorbent technique with reported detection limits: 2.0 pg/mL for RANTES and 1.7 pg/mL for CCL2. The dilution factors for both, RANTES and CCL2, were first tested in our preliminary study (data nor present). According to the manufacturer instructions, all platelet poor plasma samples for RANTES were diluted 4-fold using Calibrator Diluent RD6-11 (R&D Systems). The manufacturer of assay kit referred to the intra-assay coefficient of variation (CV%) as 2.5% at RANTES mean concentration of 91.9 pg/mL, SD = 2.30 pg/mL. According to the manufacturer's instructions, all plasma samples for CCL2 were diluted 2-fold using Calibrator Diluent RD6Q (R&D Systems). The manufacturer of assay kit referred to the intra-assay coefficient of variation (CV%) as 7.8% at CCL2 mean concentration of 76.7 pg/mL, SD = 6.0 pg/mL. The reading of the absorbance was performed on a microplatelet reader Multiskan Go (Thermo Scientific).

### 2.4. Biochemical Parameters Evaluation

In order to determine troponin I, hsCRP, total cholesterol, LDL-cholesterol, HDL-cholesterol, triglycerides, glucose, and creatinine, blood (2.6 mL) was collected into the test tubes S-Monovette (SARSTEDT) without anticoagulant. Within 2h after the venipuncture, blood was centrifuged to obtain serum samples. The above-mentioned parameters, except hsCRP, were tested on the Architect c8000 and Architect ci4100 (Abbott Diagnostics, IL, USA) analyzers. GFR was calculated with the use of a MDRD formula. hsCRP concentration was determined using immunoturbidimetry on the Cobas Integra® 400 plus (Roche Diagnostics) analyzer by means of cardiac C-reactive protein test.

### 2.5. Morphological Parameters Evaluation

WBC, PLT, and MPV were determined in the blood (2.7 mL) collected into S-Monovette EDTA-K3 tubes (SARSTEDT) on a XN 1000 (Sysmex) hematological analyzer.

### 2.6. Statistical Analysis

The obtained results were statistically analyzed with the use of the STATISTICA 12.0 PL software (StatSoft Inc., Tulsa, USA) and STATA 12.1 (StataCorp LP). The concentrations of parameters tested were not following a normal distribution in the preliminary statistical analysis (Shapiro-Wilk test); thus nonparametric statistical analysis was employed. The Mann-Whitney test was used in order to compare two independent samples, and Kruskal-Wallis test was used for the comparison of three samples. A *χ*^2^ test was used to determine whether there was a difference in hypertension, diabetes, hyperlipidemia, obesity, smoking, and medication therapy. If not stated otherwise, the values for each given measured variable are given as median and interquartile ranges (IQs).

In the group of cardiovascular disease patients (AMI + SA) we performed a univariate linear regression analysis, as well as multiple linear regression analysis, to indicate factors that may influence plasma RANTES and CCL2 concentrations. Tested factors included age, sex, white blood cells count (WBC), monocyte count, platelet count (PLT), mean platelet volume (MPV), hsCRP concentration, creatinine and eGFR value, applied treatments (aspirin, heparin, and clopidogrel), and coronary risk factors (lipid profile parameters, glucose concentration, blood pressure, heart rate, ejection fraction, obesity, and smoking). Significantly skewed variables were logarithmically transformed. Differences were considered statistically significant for P<0.05.

## 3. Results

### 3.1. Plasma RANTES and CCL2 Concentrations

The highest median RANTES concentration was observed in SA group; it was 3.4-fold higher than in control subjects (*P<0.001*). In AMI group RANTES concentration was 2.8-fold higher as compared to control group (*P<0.001*). Analysis of RANTES concentrations depending on the severity of signs and symptoms of AMI found that STEMI patients had 2.2-fold and NSTEMI 3.1-fold higher levels of protein tested, as compared to control group (*P=0.008* and* P<0.001*, respectively) (Table 3).

The highest median CCL2 concentration was observed also in SA group; it was 1.4-fold higher than in control subjects (*P=0.004*). In AMI group CCL2 concentration was almost 1.2-fold higher as compared to control group (P>0.05). STEMI patients had 1.2-fold higher while NSTEMI individuals had 1.3-fold higher levels of chemokine tested as compared to healthy group; however, none of above-mentioned differences were statistically significant (P>0.05) (Table 3).

In the next step of our analysis cardiovascular disease patients were divided into those with ≤ median value and those with > median value based on age, MPV value, chemokines, and hsCRP concentration. In AMI group we found significant differences (*P<0.05*) in RANTES concentration for patients divided depending on age, hsCRP, and CCL2 value. Similar analysis was performed for CCL2; however, we did not show any significant differences (Table 4). In SA group we did not find any differences, neither for RANTES nor for CCL2 (data not present).

### 3.2. Males Results versus Females Results

AMI males had statistically higher RANTES concentration (5464 pg/mL; IQs: 3338-7472 pg/mL) compared to females (3819 pg/mL; IQs: 1633-5551 pg/mL) (*P=0.013*). Contrary to RANTES results, CCL2 concentration in AMI males was lower (178 pg/mL; IQs: 166-202 pg/mL) compared to AMI females (209 pg/mL; IQs: 174-274 pg/mL); however obtained difference was insignificant (P>0.05).

SA males had higher RANTES concentration (5935 pg/mL; IQs: 3810-6656 pg/mL) compared to SA females (5461 pg/mL; IQs: 4256-8317 pg/mL). Similarly to AMI males, also SA males presented lower CCL2 concentration (196 pg/mL; IQs: 151-242 pg/mL) compared to SA females (227 pg/mL; IQs: 206-378 pg/mL). However, none of above-mentioned differences were of statistical significance (P>0.05).

### 3.3. Differences in RANTES and CCL2 Concentrations between 1-Vessel and Multivessel Coronary Artery Disease

Median RANTES concentration in multivessel disease subgroup of AMI patients (5552 pg/mL; IQs: 2752-7268 pg/mL) was 1.4-fold higher as compared to 1-vessel disease subgroup (3856 pg/mL; IQs: 2596-6136 pg/mL). In SA individuals multivessel disease subgroup patients had lower RANTES concentrations (5668 pg/mL; IQs: 3992-6356 pg/mL) as compared to 1-vessel disease patients (6892 pg/mL; IQs: 3328-10156 pg/mL). Median CCL2 concentration in multivessel disease subgroup of AMI patients (180 pg/mL; IQs: 166-252 pg/mL) was the same as in 1-vessel disease AMI subgroup (180 pg/mL; IQs: 170-208 pg/mL). Median CCL2 concentration in multivessel disease subgroup of SA subjects (236 pg/mL; IQs: 196-366 pg/mL) was lower than in 1-vessel disease subgroup (212 pg/mL; IQs: 178-266 pg/mL). However none of obtained differences were statistically significant (P>0.05), neither in AMI patients nor in SA group.

### 3.4. Univariate Linear Regression Analysis for RANTES in Cardiovascular Disease Patients

Univariate linear regression analysis found that for men mean RANTES plasma level is 1.56 times higher as compared to women (e^*β*^=1.563;* P=0.003*). We found that if patient's age increases by 1 year, the mean RANTES concentration value increases by 1.4% (e^*β*^=1.014;* P=0.018*). Univariate linear regression analysis also revealed that if CCL2 concentration increases by 10 pg/mL, the mean RANTES concentration value increases by 3.3% (e^*β*^=1.003;* P<0.001*). Additionally we found that if hsCRP concentration increases by 1 mg/L, the mean RANTES concentration value increases by 1.0% (e^*β*^=1.010;* P=0.002*) (Table 5).

Lipid profile parameters, WBC, monocyte count, PLT, MPV, glucose, creatinine value, coronary risk factors, and applied treatments (aspirin, clopidogrel, and heparin) did not influence plasma RANTES concentration.

### 3.5. Multiple Linear Regression Analysis for RANTES in Cardiovascular Disease Patients

In the model of multiple linear regression analysis predictor variables influencing plasma RANTES concentrations included sex, MPV, and CCL2 concentrations. The adjusted R^2^ equals 0.30, which means that the model explains 30% of the variance in dependent variable.

By means of multiple linear regression analysis we found that for men the mean plasma RANTES concentration value increases 1.89 times as compared to women (e^*β*^=1.892;* P<0.001*), if other model parameters are fixed. If CCL2 concentration increases by 10 pg/mL, the mean RANTES concentration value increases by 3.4% (e^*β*^=1.003;* P<0.001*), if other model parameters are fixed. We also found that if MPV increases by 1 fL, the mean RANTES concentration value increases by 12% (e^*β*^=1.121;* P=0.028*), if other model parameters are fixed (Table 5).

### 3.6. Linear Regression Analysis for CCL2

For CCL2 we did not obtain statistically significant linear regression analysis results, neither univariate nor multiple.

## 4. Discussion

Data concerning circulating concentrations for RANTES and CCL2 is incoherent [14–20]. In the current study we found different biomarker patterns for RANTES and CCL2 levels in AMI patients and SA individuals. Median RANTES concentration was statistically elevated both in AMI and in SA groups as compared to healthy control group. Analysis of CCL2 concentration revealed that only SA individuals had significantly higher levels of this CC chemokine as compared to control subjects. Interestingly, we found the highest concentrations for both chemokines tested in SA patients, which may indicate these proteins as biomarkers of the presence of chronic coronary artery disease rather than the acute state. Both chemokines participate in the formation of atherosclerotic plaque, although other mechanisms are also involved here. RANTES mainly derives from activated platelets and its role seems to be crucial in the initiation of atherosclerosis plaque formation, while CCL2 recruits monocytes which contribute to the progression of atherosclerotic lesion.

In our study we observed that the median RANTES concentration was several times higher (approximately 3-fold) in patients with both AMI and SA, as compared to those in the control group, whereas the median CCL2 was only slightly higher in both groups of patients with cardiovascular disease compared to control subjects, which may indicate that the circulating RANTES concentration reflects the presence of the atherosclerotic lesions better than CCL2.

It was found that the total wall volume, maximum wall thickness, vessel wall area, and mean minimum fibrous cap thickness were positively associated with RANTES concentration [8]. It was also proved that RANTES antagonists inhibit monocyte recruitment on injured carotid endothelium and atherosclerosis progression as well as preventing SA [5, 21, 22]. Therefore, in the next step of our analysis we tried to find out if RANTES or CCL2 concentrations are influenced by the advancement of coronary artery changes. For both chemokines tested we did not reveal significant differences between 1-vessel disease and multivessel disease, neither in AMI patients nor in SA group. Our findings are in line with the results of Podolec et al. [23] and Nishiyama et al. [24], who also did not reveal significant association between RANTES and CCL2 concentrations and the number of coronary vessels involved, respectively. We hypothesize that RANTES and CCL2 circulating levels are rather biomarkers of the presence of atherosclerotic lesion, than the markers of its severity.

Some authors indicated elevated RANTES concentrations in patients with AMI, as compared to healthy control group, but chemokine levels did not differ between patients with SA and the healthy group [15]. On the other hand, Cavusoglu et al. [16] reported that in males with known or suspected coronary artery disease a low baseline RANTES concentration was an independent predictor of myocardial infarction and cardiac mortality. Moreover, our results are in disagreement with the previous findings. These controversies may result from the fact that RANTES levels present significant ethnic variations, as Virani et al. [8] revealed the highest RANTES concentrations in Caucasian females, followed by Caucasian males, African-American females, and African-American males. However, in our study population, we found that Caucasian males had higher RANTES concentrations as compared to Caucasian females. Despite the fact that our study group and Virani's group were from different geographical regions (Europe* vs. *US), both were Caucasian. Interestingly, the data concerning the distribution of RANTES promoter-28>G and -403G>A gene polymorphisms is incoherent, which may indicate the potential source of variability in RANTES circulating concentrations [17–19]. Nevertheless, the quantitative RANTES evaluation in cardiovascular disease patients requires further investigations.

As it was mentioned above, CCL2 levels were higher in both AMI group and SA individuals as compared to control group; however, statistically significant differences were found only between SA subjects and healthy subjects. Our results are in disagreement with the findings of Arakelyan et al. [20], who revealed significantly elevated CCL2 concentrations in AMI group as compared to the control group. However, there are some differences regarding the risk factors between these two studies: (1) 58% of the participants included in our AMI group had hypertension versus 44% of Arakelyan et al. [20] group; (2) 25% of the participants included in our AMI group had diabetes versus 18% of Arakelyan et al. [20] group. Previous studies found that CCL2 levels are associated with hypertension and diabetes [25, 26]. Moreover, contrary to Arakelyan et al. [20], we tested CCL2 concentrations in plasma samples not serum and we included more females in the study group (25* vs.* 8).

On the other hand, the results of Murakami et al. [14] are in line with ours, as they also did not find differences for CCL2 between AMI patients and healthy controls. Interestingly Murakami et al. [14] found that CCL2 levels significantly increased after 7 days of myocardial infarction. Also, Economou et al. [27] revealed that CCL2 concentrations significantly rose after 3 and 6 months following percutaneous coronary transluminal angioplasty (PTCA). We tested CCL2 concentrations on patient's admission to the hospital. Therefore, the time aspect of sample collection to the quantitative evaluation of CCL2 concentrations for further clinical interpretation is important.

Study of de Lemos et al. [26] on a large cohort of acute coronary syndrome patients showed that an elevated baseline CCL2 concentration was related to traditional risk factors for atherosclerosis, as well as to an increased risk of death after myocardial infarction. CCL2 is best known for the chemotactic properties of monocytes and involvement in atherosclerosis development. Secreted by endothelial cells, it binds to heparin sulphate on monocytes surface to form oligomers and thus forms a key point for its receptor CCR2 found on circulating monocytes [28, 29]. This promotes the attachment of monocytes to endothelial cells and their further migration. If CCL2 is secreted by cells at the site of ongoing inflammation, it induces chemotaxis in this area. Migration of inflammation cells is initiated by the release of matrix metalloproteinases (MMPs) by leukocytes that mark the “pathway” through endothelial cells. MMPs have no effect on CCL2. However, when the inflammatory cells reach the target site, other metalloproteinases such as MMP-1 and MMP-3 inactivate CCL2 causing inhibition of the inflammatory process [30]. It is also suggested that CCL2 may have influence on a number of processes occurring in the course of myocardial infarction, such as myocardial necrosis, apoptosis, leukocyte recruitment, treatment of myocardium, and formation of scars, as well as angiogenesis [1, 31]. We found increased CCL2 levels both in AMI and SA individuals compared to control group, which may result from the above-mentioned CCL2 roles in the pathophysiology of cardiovascular diseases.

In our study group we did not find statistical differences for CCL2 plasma concentrations between AMI patients and SA individuals, which is in line with findings of other authors [27]. Moreover, Kameda et al. [32] did not find significant differences for CCL2 concentrations measured in pericardial fluid of AMI patients and angina pectoris group. These findings altogether may indicate association between CCL2 and pathological conditions present in early (atherosclerosis, acute ischemia) and late (reperfusion injury, fibrosis remodeling, heart failure, cardiac arrhythmias) cardiovascular diseases stages [31].

In the next step of our analysis we tried to establish factors that may influence plasma RANTES or CCL2 concentration. For CCL2 we did not obtain statistically significant linear regression models, neither univariate nor multivariate, probably due to high variability of obtained concentrations for protein tested.

In the model of multiple linear regression analysis predictor variables influencing plasma RANTES concentration included sex, MPV, and CCL2 concentration. Studies of Blanchet et al. [33] indicated that elevating heparin doses added to plasma* in vitro* increases RANTES concentration. However, both our and Blanchet et al.'s [33] multiple linear regression analysis results excluded heparin as a relevant factor influencing RANTES plasma concentration in cardiovascular disease patients. Interestingly, in our study population, other applied treatments (aspirin and clopidogrel) did not influence plasma RANTES levels.

Contrary to Jang et al. [17], we did not find correlation of RANTES with platelet count; however, we revealed a correlation of chemokine tested with MPV, which is a well-established biomarker of platelet activation [34]. These discrepancies may be related to different ethnic groups analyzed (Korean* vs.* Caucasian), different materials evaluated (poor platelet plasma* vs.* serum), and the fact that Jang et al. [17] tested only males, while our study group included both sexes. RANTES is stored in platelet vesicles and released upon platelet activation. The positive correlation of RANTES with MPV may indicate that circulating concentration of chemokine tested is not related to the platelet count but rather to platelet hyperactive state. RANTES is stored in intracellular platelets alpha granules in the basal state and it is released upon their activation [8, 19]. Therefore, to measure circulating plasma RANTES concentration, complete platelet removal from blood is needed, which indicates that more attention should be paid to the appropriate process of plasma centrifugation. We measured RANTES concentration in platelet poor plasma, which excludes the possibility of increased RANTES concentration due to* in vitro* platelet release during sample processing.

In our study only the unadjusted model showed that RANTES levels are influenced by age, which contradicts the results of Ueba et al. [35] as they found, in both univariate and multivariate linear regression analysis, that age was one of the most significant factors influencing RANTES concentrations, but their studies were conducted on healthy Japanese males.

In univariate linear regression analysis, we revealed that hsCRP concentration influences RANTES levels, which is also contrary to Ueba et al.'s [35] findings. This may be explained by the fact that RANTES activates expression of cytokines which in turn lead to activation of CRP synthesis by hepatocytes [17]. The study group of Ueba et al. [35] was composed of healthy younger men (mean age 41 years old), with no inflammatory state as well as signs, symptoms, or a history of cardiovascular disease. According to Blanchet et al. [33], the relationship of RANTES with C-reactive protein concentration highlights its mediating role in the inflammatory processes. They also showed, using multiple linear regression analysis, that CRP is a significant factor associated with RANTES levels.

The limitation of the present study is a small patient population, so there could be a selection bias (type II error). Secondly, the study should be developed on the CCL2 and RANTES genetic polymorphisms evaluation in polish cardiovascular diseases population, as genetical variations can influence the chemokines circulating concentration levels [17–19]. We have designed such a study and are currently in the process of applying for funds. Another limitation of our study may result from the preanalytical variables, which may influence plasma circulating chemokines levels. It is well established that MPV is a marker of platelet activity [36] and RANTES is stored in platelets granules and released upon their activation [8, 19]. In our study we found a positive relation of RANTES and MPV suggesting that such factors like lifestyle (including diet), physical activity, alcohol intake, and hormonal profile, as well as genetic factors, may influence MPV [36, 37], which may consequently lead to the increased platelet activity and thus plasma RANTES concentration. It should be also noted that most CC chemokines are able to form oligomers to form higher-small-molecular-weight (MW) complexes in the presence of glycosaminoglycans (GAGs)* in vitro* [38]. However, available literature indicates that the CC chemokines oligomerization also may occur* in vivo* [39–41], and these CC oligomers are resistant to proteases contrary to monomeric CC chemokines that are susceptible to proteolysis [38]. Finally, our study was only retrospective, as it examined RANTES and CCL2 levels only at hospitalization. Kraaijeveld et al. [42] showed increased RANTES concentration in unstable angina pectoris (UAP) patients compared to stable angina pectoris patients at baseline (at the time of hospitalization), which significantly decreased within 180 days after the UAP symptoms had occurred. Their findings indicate the necessity of prospective study design. Therefore the validation of factors associated with RANTES and CCL2 circulating concentrations in cardiovascular disease patients requires further studies, taking into account the above-mentioned issues.

## 5. Conclusions

RANTES and CCL2 levels were higher in SA patients compared to AMI individuals, which may indicate these proteins as biomarkers of the presence of chronic coronary artery disease rather than the acute state. However presented chemokines do not reflect the atherosclerotic lesion severity, as we did not show differences for both RANTES and CCL2 between 1-vessel and multivessel coronary artery disease. Due to high variability of obtained CCL2 concentrations, it seems that RANTES reflects the presence of the atherosclerotic lesion better than CCL2. Circulating RANTES concentration should be interpreted depending on patient's sex, age, platelet hyperactivity state, and hsCRP as well as CCL2 concentration.

The potential clinical implications of our study are a better understanding of factors that may be associated with RANTES circulating concentration, owing to the fact that the inhibition of chemokine-mediated recruitment and activation of monocytes may present a novel therapeutic target to counteract the development and progression of atherosclerotic plaque formation.

## Figures and Tables

**Table 1:** Clinical characteristics of AMI subjects depending on the severity of signs and symptoms (STEMI, NSTEMI) as compared to SA individuals. Results are presented as median and interquartiles.

	AMI N=83	STEMI N=38	NSTEMI N=45	SA N=32	AMI *vs.* SA STEMI *vs.* SA NSTEMI *vs.* SA	STEMI *vs.* NSTEMI
Systolic BP [mmHg]	140 (126-151)	140 (126-150)	136 (126-153)	129 (100-145)	NS NS NS	NS
Diastolic BP [mmHg]	85 (77-92)	88 (80-96)	80 (75-90)	76 (55-90)	NS NS NS	NS
Ejection fraction [%]	45 (40-55)	45 (43-50)	48 (38-60)	55 (28-60)	NS NS NS	NS
Heart rate/minute	75 (66-88)	80 (70-90)	74 (65-87)	90 (68-150)	NS NS NS	NS
*Medications on admission*						
Aspirin N (%)	78 (94%)	37 (97%)	41 (91%)	4 (13%)	*<0.001* *<0.001* *<0.001*	NS
Clopidogrel N (%)	64 (77%)	29 (76%)	35 (78%)	1 (3%)	*<0.001* *<0.001* *<0.001*	NS
Heparin N (%)	13 (16%)	4 (11%)	9 (20%)	-	*0.017* NS *0.027*	NS
*Coronary risk factors*						
Hypertension N (%)	58 (70%)	29 (76%)	29 (64%)	20 (63%)	NS NS NS	NS
Diabetes t.2 N (%)	25 (30%)	14 (37%)	11 (24%)	12 (38%)	NS NS NS	NS
Hyperlipidemia N (%)	53 (64%)	25 (66%)	28 (62%)	15 (47%)	NS NS NS	NS
Obesity N (%)	22 (27%)	13 (34%)	9 (20%)	3 (9%)	*0.045* *0.013* NS	NS
Smoking N (%)	21 (25%)	10 (26%)	11 (24%)	1 (3%)	*0.006* *0.007* *0.011*	NS

AMI: acute myocardial infarction, BP: blood pressure, N: number of individuals, NS: not statistically significant, NSTEMI: non-ST elevation myocardial infarction, SA: stable angina, STEMI: ST elevation myocardial infarction.

**Table 2:** Laboratory parameters of cardiovascular disease patients compared to control group. Results are presented as median and interquartiles.

	AMI N=83	STEMI N=38	NSTEMI N=45	SA N=32	C N=25	AMI *vs.* SA STEMI *vs.* SA NSTEMI *vs.* SA	STEMI *vs.* NSTEMI	AMI vs. C STEMI vs. C NSTEMI vs. C SA vs. C
Sex: M/F	58/25	25/13	33/12	27/5	17/8	-	-	-
Age (years)	67 (61-78)	65 (57-78)	70 (63-77)	68 (61-73)	66 (52-74)	NS NS NS	NS	NS NS NS NS
*Laboratory parameters on admission*								
WBC [x10^3^/*µ*L]	8.80 (7.84-10.64)	9.80 (8.3-12.5)	8.30 (7.08-10.06)	6.96 (6-18-8.55)	6.00 (4.99-6.49)	*<0.001* *<0.001* NS	NS	*<0.001* *<0.001* *<0.001* * 0.011*
Monocyte count [x10^3^/*µ*L]	0.78 (0.55-0.90)	0.79 (0.56-0.96)	0.76 (0.55-0.89)	0.61 (0.51-0.75)	0.50 (0.45-0.67)	*0.029* NS NS	NS	*0.002* *0.004* *0.006* NS
PLT [x10^3^/*µ*L]	212 (183-245)	210 (186-246)	212 (182-245)	211 (174-254)	236 (222-246)	NS NS NS	NS	NS NS NS NS
MPV [fL]	10.7 (9.6-11.5)	10.7 (9.3-11.7)	10.8 (9.7-11.3)	11.5 (10.9-11.9)	10.9 (10.0-11.3)	*0.002* *0.029* *0.010*	NS	NS NS NS NS
hsCRP [mg/L]	7.40 (2.90-19.10)	5.10 (2.70-13.00)	8.90 (3.10-21.30)	1.65 (0.90-4.50)	-	*0.002* NS *0.002*	NS	-
Total cholesterol [mg/dL]	181 (147-220)	183 (166-219)	175 (146-225)	142 (133-172)	192 (169-235)	*<0.001* *<0.001* *0.017*	NS	NS NS NS *<0.001*
LDL-cholesterol [mg/dL]	116 (90-145)	117 (100-142)	114 (80-153)	87 (75-113)	133 (100-171)	*0.005* *0.015* NS	NS	NS NS NS *0.001*
HDL-cholesterol [mg/dL]	41 (34-46)	43 (37-47)	39 (33-46)	40 (35-48)	57 (49-65)	NS NS NS	NS	*<0.001* *<0.001* *<0.001* *<0.001*
TG [mg/dL]	123 (86-176)	113 (89-152)	127 (84-182)	104 (95-152)	108 (83-120)	NS NS NS	NS	NS NS NS NS
Glucose [mg/dL]	109 (96-138)	120 (103-164)	107 (95-119)	100 (88-112)	96 (90-104)	*0.018* *0.008* NS	NS	*0,001* * <0.001* *0.020* NS
Creatinine [mg/dL]	0.94 (0.81-1.13)	0.98 (0.82-1.11)	0.90 (0.81-1.21)	0.95 (0.82-1.14)	0.76 (0.72-0.90)	NS NS NS	NS	*0.003* *0.008* *0.006* *0.001*
eGFR [mL/min/1.73 m^2^]	81 (62-95)	80 (61-99)	82 (63-95)	83 (66-92)	91 (79-107)	NS NS NS	NS	*0.013* *0.025* *0.025* *0.021*

AMI: acute myocardial infarction, eGFR: estimated glomerular filtration rate, F: female, hsCRP: high sensitivity C-reactive protein, M: male, MPV: mean platelet volume, N: number of individuals, NS: not statistically significant, NSTEMI: non-ST elevation myocardial infarction, PLT: platelet count, SA: stable angina, STEMI: ST elevation myocardial infarction, TG: triglycerides, WBC: white blood cells count.

*Conversion factors to SI units are as follows:* for WBC: 1.0, for total cholesterol: 0.0259, for LDL-cholesterol: 0.0259, for HDL-cholesterol: 0.0259, for TG: 0.0114, for glucose: 0.0555, for creatinine: 88.402.

**Table 3:** RANTES and CCL2 results in AMI patients, SA subjects, and control group. Results are presented as median and interquartiles.

PARAMETER	AMI	STEMI	NSTEMI	SA	C	AMI *vs.* SA STEMI *vs.* SA NSTEMI *vs.* SA	STEMI *vs.* NSTEMI	AMI vs. C STEMI *vs.* C NSTEMI *vs.* C SA vs. C
RANTES [pg/mL]	4800 (2596-7244)	3820 (1992-6292)	5412 (3856-8104)	5884 (3808-6684)	1724 (1332-2564)	NS NS NS	NS	*<0.001* *0.008* *<0.001* *<0.001*

CCL2 [pg/mL]	180 (168-226)	176 (160-210)	190 (172-246)	210 (184-266)	152 (114-206)	NS NS NS	NS	NS NS NS *0.004*

AMI: acute myocardial infarction, CCL2: Monocyte Chemotactic Protein 1, NS: not statistically significant, NSTEMI: non-ST elevation myocardial infarction, RANTES: Regulated upon Activation, Normal T cell Expressed and presumably Secreted, SA: stable angina, STEMI: ST elevation myocardial infarction.

**Table 4:** RANTES and CCL2 concentration in AMI group based on median value of age, MPV, chemokines, and hsCRP concentration.

AGE [years]			
	≤ 67	> 67	P-value
RANTES [pg/mL]	4343 (1993-6293)	5565 (3536-8515)	*0.048*
CCL2 [pg/mL]	180 (169-226)	180 (165-246)	NS

hsCRP [mg/L]			
	≤ 7.4	> 7.4	

RANTES [pg/mL]	4157 (2214-6181)	6063 (3487-8390)	*0.048*
CCL2 [pg/mL]	192 (173-225)	173 (163-202)	NS

MPV [fL]			
	≤ 10.7	> 10.7	

RANTES [pg/mL]	5369 (1993-7470)	4551 (2753-6952)	NS
CCL2 [pg/mL]	190 (172-258)	171 (157-203)	NS

CCL2 [pg/mL]			
	≤ 180	> 180	

RANTES [pg/mL]	3617 (1633-6135)	6063 (2199-9054)	NS

RANTES [pg/mL]			
	≤ 4800	> 4800 [pg/mL]	

CCL2 [pg/mL]	173 (164-200)	198 (174-256)	*0.036*

CCL2: Monocyte Chemotactic Protein 1, hsCRP: high sensitivity C-reactive protein, MPV: mean platelet volume, NS: not statistically significant, RANTES: Regulated upon Activation, Normal T cell Expressed and presumably Secreted.

**Table 5:** Univariate and multivariate linear regression analysis results for logarithm of RANTES.

		Univariate linear regression analysis		
No	Covariate	*β*	e^*β*^ (95%CI)	P-value
1	Sex	0.447	1.563 (1.16-2.10)	0.003
2	Age	0.014	1.014 (1.00-1.02)	0.018
3	CCL2 [pg/mL]	0.003	1.003 (1.00-1.01)	<0.001
4	hsCRP [mg/L]	0.010	1.010 (1.00-1.02)	0.002

		Multivariate linear regression analysis		
No	Covariate	*β*	e^*β*^ (95%CI )	P-value

1	Sex	0.638	1.892(1.36-2.63)	<0.001
2	CCL2 [pg/mL]	0.003	1.003 (1.00-1.00)	<0.001
3	MPV [fL]	0.114	1.121 (1.01-1.24)	0.028

CI: Confidence Interval, CCL2: Monocyte Chemotactic Protein 1, hsCRP: high sensitivity C-reactive protein, MPV: mean platelet volume, RANTES: Regulated upon Activation, Normal T cell Expressed and presumably Secreted.

## Data Availability

The datasets generated and analyzed during the current study are not publicly available but all are kept at the Medical University of Bialystok and available from the corresponding author (Olga M. Koper-Lenkiewicz) on reasonable request.
